# The challenges of muscle biopsy in a community based geriatric population

**DOI:** 10.1186/s13104-018-3947-8

**Published:** 2018-11-26

**Authors:** Daisy Wilson, Leigh Breen, Janet M. Lord, Elizabeth Sapey

**Affiliations:** MRC-ARUK Centre for Musculoskeletal Ageing Research, Institute of Inflammation and Ageing, University of Birmingham, Queen Elizabeth Hospital Birmingham, Edgbaston, Birmingham, B15 2WD UK

**Keywords:** Sarcopenia, Frailty, Bergstrom needle, Percutaneous muscle biopsy

## Abstract

**Objectives:**

To describe the difficulties of obtaining muscle samples using a Bergstrom needle technique in a frail older adult population. The data were obtained from a study primarily investigating immunosenescence in frailty. An intended research technique was skeletal muscle biopsy in a small subset of participants to investigate muscle morphology and local inflammatory factors.

**Results:**

Forty healthy older adults and 37 frail older adults were considered for a Bergstrom needle muscle biopsy. Of these, 17.5% of healthy older adults and 94.6% of the frail older adults had single or multiple participant factors resulting in a contra-indication to muscle biopsy. 40.7% of healthy older female participants were at risk of a failed muscle biopsy due to low muscle mass. Considering only muscle mass muscle biopsy would have been successful in 18.7% of the frail older women and 21.4% of the frail older men. In this population, muscle biopsy was not feasible because of contra-indications in the majority of participants. This questions whether a biopsy sample obtained from frail older individuals, is actually representative of this population and supports the need to disclose biopsy failure rate in this population.

**Electronic supplementary material:**

The online version of this article (10.1186/s13104-018-3947-8) contains supplementary material, which is available to authorized users.

## Introduction

Sarcopenia, the loss of muscle mass and physical function, and frailty, a syndrome of diminished strength, endurance, and reduced physiological function that increases the risk of adverse outcomes, are both common conditions affecting older adults [1, 2]. The syndromes although separate conditions share several characteristics and often co-exist in the same individual, the relationship between the two conditions is complex and poorly defined [3]; these reviews address these aspects in greater detail [4, 5]. The pathophysiology of both syndromes is complex, however, reduced muscle health is central to both conditions [6]. ‘Muscle quality’, including morphological characteristics of the muscle, aerobic capacity, intramuscular adipose tissue, fibrous tissue and motor units [7], is considered to be as, or more, important than muscle mass in the development of sarcopenia and frailty.

Investigators require muscle tissue to investigate factors contributing to muscle quality. Although animal models exist, the ability of these models to recapitulate the complexity and heterogeneity of both syndromes is limited [8], necessitating the use of human tissue. Muscle tissue from humans can be obtained from either a percutaneous muscle biopsy, preferred for most research, or as a by-product of surgery, where the sample may be altered by the surgery itself rather than the underlying condition. Percutaneous muscle biopsy has been used with success on multiple occasions in individuals with sarcopenia [9–12] and on fewer occasions in frailty [13]. A recent study reported on both the acceptability and feasibility of muscle biopsy in an older community-dwelling male population and highlighted the importance of completing this procedure in a frail population [11]. Ultrasound guidance for the prior assessment of muscle thickness and site choice is not mandated for muscle biopsy [14], but is becoming increasingly popular with researchers [15, 16].

This paper reports on the difficulties of obtaining muscle samples using a percutaneous Bergstrom needle technique in a frail older adult population [14] and potential problems in obtaining reasonable muscle sample sizes in both healthy older adults and frail older adults. The data were obtained from a study primarily investigating immunosenescence in frailty. An intended research technique was muscle biopsy in a small subset of participants (N = 5) to obtain muscle samples to investigate muscle morphology and local inflammatory factors.

## Main text

### Study design

Forty healthy older adults (aged > 65 years, no chronic inflammatory disease, malignancy or immunosuppressive medications) and 37 frail older adults (> 65, frailty index > 0.2 [17], able to provide written consent, no malignancy or immunosuppressive medications) were recruited from the community (100% healthy older adults; 48.6% frail older adults) or a “medically stable for discharge” inpatient population (51.4% frail older adults). Table 1 illustrates the demographics of the cohorts.

**Table 1:** Demographic data of recruited participants

	Healthy older adults	Frail older adults	Statistics
Gender	67.5% (27) female	54.1% (20) female	p = 0.241
	32.5% (13) male	45.9% (17) male	
Age	71.9 ± 9.0	84.0 ± 15.0	p < 0.000
BMI	23.4 ± 4.0	24.9 ± 7.0	p = 0.782
FI	0.03 ± 0.07	0.41 ± 0.17	p < 0.000

Data sets: healthy older adults, n = 40; frail older adults, n = 37. Median ± IQR are given. Categorical data are given as percentage of total population with raw number in brackets. Categorical data tested with Pearson’s Chi Squared and continuous data tested with Mann–Whitney U. All data not normally distributed except frail older age and frail older frailty index (FI). Statistical difference analysed with independent samples Mann–Whitney U

All individuals underwent assessment of frailty [17] and sarcopenia [2] including an ultrasound to determine quadriceps muscle thickness bilaterally and identify the leg with thickest muscle and therefore most suitable for biopsy.

During recruitment it was evident that acquiring a sufficient number of muscle biopsy samples would be challenging, due to participant factor contra-indications to muscle biopsies (discussed in “Participant factors” section) in frail older adults, and concerns about how representative any frail older adult muscle samples would be in comparison to the frail older adult cohort as a whole. Therefore, this line of investigation was abandoned before any muscle samples were taken in either healthy older or frail older adults.

The muscle biopsies were planned to be performed in an outpatient setting following a single prior visit for safety bloods [platelets and international normalised ratio (INR)]. The muscle biopsies were to be taken from the vastus lateralis with a percutaneous method using a Bergstrom needle [14]. This method was chosen due to local expertise and success in community-based healthy older adults in an inpatient setting [12, 18].

The data presented illustrate the contra-indications to muscle biopsies described as participant factors and muscle thickness.

### Participant factors

Participant factors resulting in contra-indications to muscle biopsy were categorised as: safety of biopsy, ability to attend for biopsy, ability to care for wound post-biopsy and anticipated high risk for complications. Examples for each category are described in Additional file 1: Table S1 and are specific to this sample.

Clinical guidelines suggest muscle biopsies are not performed on individuals on anti-platelet or anti-coagulation therapy [19]; all other contra-indications are proposed on pragmatic grounds and were considered by two experienced geriatricians prior to the individual’s exclusion from the muscle biopsy sub-study. Relative contra-indications are factors which could be mitigated with appropriate resources, such as daily nursing care.

**Fig. 1:**
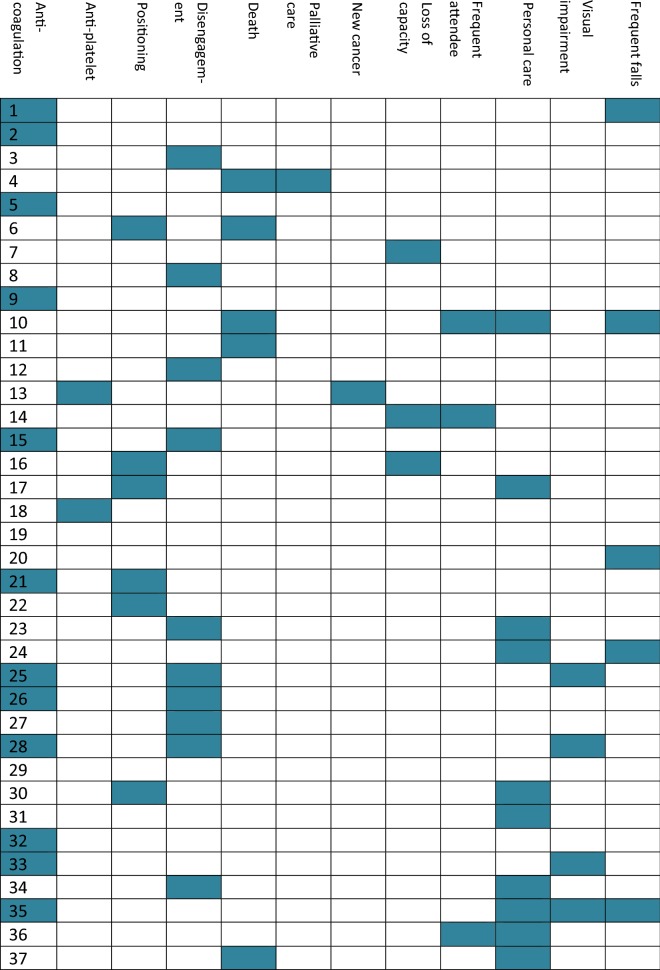
Frequency of contra-indications to muscle biopsy in the frail older adults. Each row represents a single frail older individual. Each column represents the contra-indication. Coloured block represents that individual having that contra-indication

17.5% healthy older adults had a recognised contra-indication to muscle biopsy. All these participants were precluded from undergoing a muscle biopsy due to the safety of biopsy, secondary to anti-platelet or anti-coagulant use. The majority of frail older adults had more than one contra-indication to muscle biopsy: 32.4% had a single contra-indication, 40.5% had two, 13.5% had three, 5.4% had four and 2.7% had five contra-indications. Inability to attend (54.1%), due to death, disengagement with services and loss of capacity, and safety of the biopsy (51.3%) were the most frequent contra-indications. Therefore, only 5.4% of the frail older adults did not have a contra-indication to muscle biopsy. Figure 1 demonstrates both the frequency of each contra-indication and the number of participants with multiple contra-indications illustrating the complexities of the individuals.

Although these data relate to muscle biopsies performed using the Bergstrom technique, it is also applicable to other percutaneous muscle biopsy techniques, conchotome forceps and microbiopsy needle techniques [20, 21]. The contra-indications are the same for all the techniques, although it could be argued that the incision with the microbiopsy needle technique is smaller and, therefore, would reduce the burden of post-biopsy care on the individual. Nevertheless, even if this was the case only 10.8% of the frail older adult population would have no contra-indications to muscle biopsy.

### Muscle thickness

To obtain an adequate muscle sample total muscle thickness of the vastus lateralis and underlying vastus intermedius at the site of sampling should be thicker than the sum of the needle point (9.5 mm) and window (8.0 mm); the total depth of needle point and window of a Bergstrom needle is 17.5 mm. Therefore, at a muscle thickness greater than 17.5 mm, it is possible to obtain a muscle sample between 25 and 140 mg, which is generally adequate for most laboratory-based analyses of muscle morphology/biochemistry [19]. At a muscle thickness between 9.5 mm and 17.5 mm a muscle sample should be obtained, but may take multiple passes, and may not be of sufficient size for experiments and will likely contain subcutaneous adipose tissue. At a muscle thickness less than 9.5 mm, no muscle sample will be obtained. Whilst there are no reports in the literature of directly measured muscle depths linked to failed Bergstrom needle muscle biopsies, there are multiple reports of failed needle biopsies in patients with significantly wasted muscle [15, 20, 22].

Muscle thickness of the rectus femoris and vastus intermedius at 50% of the femur length (identical thickness to vastus lateralis and vastus intermedius at maximum cross-sectional area of vastus intermedius, location of muscle biopsy site [23]) was measured bilaterally in 40 healthy older and 30 frail older adults using ultrasound [Acuson Antares Premium Edition (Siemens)]. Patients were divided into those with adequate (> 17.5 mm), sub-optimal (9.5–17.5 mm) or inadequate (< 9.5 mm) muscle thickness on either leg.

**Fig. 2:**
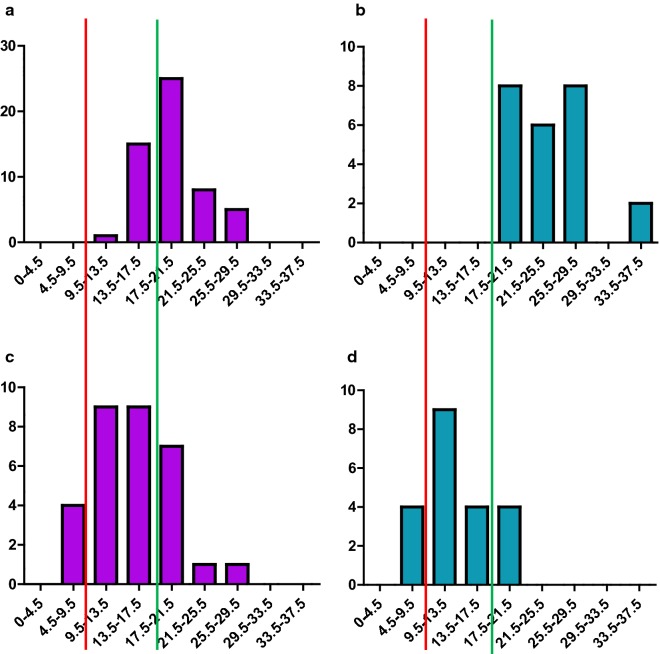
Distribution of muscle thickness in healthy and frail older adults. Distribution of muscle thickness: **a** healthy older women, **b** healthy older men, **c** frail older women, **d** frail older men. X axis is muscle thickness measured in mm. Y axis is frequency of individuals. Left of the red line are individuals in whom no muscle would be obtained using a Bergstrom needle biopsy technique. Left of the green line are individuals who are at risk of inadequate muscle samples using a Bergstrom needle biopsy technique

Analysing the entire sample (including those with a contra-indication to muscle biopsy) there was adequate muscle thickness in all healthy older males and 59.3% of the healthy older females. Figure 2 demonstrates that although there was a risk of sub-optimal sized muscle biopsy in 40.7% of healthy older females, the muscle samples obtained from these individuals was likely to be sufficient for analysis because the majority of healthy older females had muscle thicknesses closer to 17.5 mm than 9.5 mm.

In frail older adults only 18.7% of females and 21.4% of males had adequate muscle thickness for biopsy sampling. 68.8% of frail older females and 57.1% of frail older males had sub-optimal muscle thickness, suggesting multiple passes might be required to obtain an adequate sample, and 12.5% of frail older females and 21.4% of frail older males had inadequate muscle thickness. In the frail older adults with sub-optimal muscle thickness, there was a greater proportion of individuals with muscle thickness between 9.5 mm and 13.5 mm suggesting a higher chance of a failed muscle biopsy due to inadequate sample size.

## Discussion

Previous studies have suggested that muscle biopsy is a feasible tool in a frail or sarcopenic older population [9–11, 13]. To our knowledge this is the first study to demonstrate the difficulties of performing percutaneous muscle biopsies in frail older adults and we describe contra-indications in the majority of frail older adults in our cohort. Potential reasons for these reported differences in findings, include study design and the criteria employed to diagnose sarcopenia. Successful studies involving biopsy investigations approached hundreds more individuals to the study than were eventually recruited, with muscle biopsies performed in 6.3% of approached pre-frail or frail individuals [13] and 9.7% of approached community-dwelling men, of whom only 6% had sarcopenia [11]. Other successful studies diagnosed sarcopenia without assessment of muscle strength or physical performance, and it is therefore unclear whether these individuals had just low muscle mass or sarcopenia [9, 10]. In addition, a single study used criteria [appendicular lean mass (ALM)/body mass index (BMI)], which over identify sarcopenia in populations with high BMI, in a sample population with a mean BMI of 34.0 [9].

Alternative methods of obtaining muscle samples include the microbiopsy needle technique and the conchotome forceps. The microbiopsy needle is narrower, but to compensate, the window for sample collection is longer (19 mm) and, therefore, in this sample population only adequate muscle biopsy samples would be obtained at first pass in 3.7% of healthy older women, 23.1% of healthy older men and none of the frail older adults. The conchotome forceps are an alternative to a needle biopsy technique, with the advantage of the researcher being able to operate at shallower depths of muscle [19]. However, this is at the expense of increased trauma to the area with an incision 5–10 mm in length required to access the muscle.

We provide evidence that ultrasound assessment of the biopsy site prior to needle insertion should be considered in all frail populations and female community-dwelling older adults. The data presented in this chapter are contrary to current literature, which suggests that muscle biopsy is feasible in older adults with frailty and/or sarcopenia [9–11, 13]. In our cohort, the frail older participant who could undertake muscle biopsy was not representative of the group as a whole, introducing the potential for bias [younger male, aged 75 (average age in frail older adults 84.0); less frail, FI 0.33 (average FI 0.41)]. The paucity of negative results in the published literature means that whilst muscle biopsy has been possible on occasion in these populations, it is unclear whether the data presented here are an anomaly or represent a common but under-reported research experience. This does suggest that if muscle samples are fundamental to the research outcomes then the study should be designed to approach a large cohort of frail adults. Further research investigating challenges of muscle biopsy in this population is essential and should include data on acceptability of the technique.

### Study limitations

- Muscle biopsy in frail older adults was not the designed primary end-point. Successful muscle biopsy studies in frail older adults have approached and screened much larger cohorts [11, 13].
- Muscle thickness was not measured at site of biopsy but at proxy site reported to be of identical thickness [23].
- Willingness to undergo a muscle biopsy was not formally recorded but anecdotally only a small proportion of the frail older participants were agreeable. This is contrary to previous literature [11, 13].

## Additional file

**Additional file 1.** Participant factors, both absolute and relative contra-indications. Table of participant factors present in the frail older adult population that are contra-indications to muscle biopsy. Absolute contra-indications cannot be ameliorated. Relative contra-indications could be ameliorated with appropriate resources.

## Abbreviations

ALM: appendicular lean mass
BMI: body mass index
INR: international normalised ratio
