# Supplementary material for: The challenges of muscle biopsy in a community based geriatric population
Source: BMC Res Notes. 2018 Nov 26;11:830. doi: 10.1186/s13104-018-3947-8 (PMC6260758; doi:10.1186/s13104-018-3947-8)
Supplement: Supplementary file 1 — Additional file 1. Participant factors, both absolute and relative contra-indications. Table of participant factors present in the frail older adult population that are contra-indications to muscle biopsy. Absolute contra-indications cannot be ameliorated. Relative contra-indications could be ameliorated with appropriate resources. [file 13104_2018_3947_MOESM1_ESM.docx]

**Additional Table S1: Participant factors, both absolute and relative contra-indications.**

| **Participant Factors** | **Absolute Contra-Indications** | **Relative Contra-Indications** |
| --- | --- | --- |
| Safety of biopsy | - Anti-coagulation (15) - Anti-platelet (15) - Anticipated difficulties with positioning for biopsy |  |
| Ability to attend for a biopsy | - Disengagement with services/research study - Death - Started on palliative treatment - New diagnosis of cancer - Loss of capacity - Frequent attendances at hospital |  |
| Ability to care for wound post-biopsy |  | - Unable to complete personal care - Visual impairment |
| Anticipated high risk for complications | - Multiple falls |  |

Participant factors present in the frail older adult population that are contra-indications to muscle biopsy. Absolute contra-indications cannot be ameliorated. Relative contra-indications could be ameliorated with appropriate resources. The examples were all present in at least one participant.
